# The potential prolonged effect at one-year follow-up after 18-month randomized controlled trial of a 90 g/day low-carbohydrate diet in patients with type 2 diabetes

**DOI:** 10.1038/s41387-022-00193-4

**Published:** 2022-04-09

**Authors:** Chin-Ying Chen, Wei-Sheng Huang, Ming-Hua Ho, Chin-Hao Chang, Long-Teng Lee, Heng-Shuen Chen, Yow-Der Kang, Wei-Chu Chie, Chyi-Feng Jan, Wei-Dean Wang, Jaw-Shiun Tsai

**Affiliations:** 1 Department of Family Medicine, National Taiwan University Hospital & National Taiwan University, Taipei, Taiwan; 2 Department of Dietetics, National Taiwan University Hospital & National Taiwan University, Taipei, Taiwan; 3 Department of Medical Research, National Taiwan University Hospital & National Taiwan University, Taipei, Taiwan; 4 Institute of Epidemiology and Preventive Medicine, Department of Public Health, College of Public Health, National Taiwan University, Taipei, Taiwan

**Keywords:** Metabolism, Nutrition, Type 2 diabetes

## Abstract

**Objectives:**

To evaluate the effect at a one-year follow-up after an 18-month randomized controlled trial (RCT) of 90 gm/day low-carbohydrate diet (LCD) in type 2 diabetes.

**Research design and methods:**

Eighty-five poorly controlled type 2 diabetic patients with an initial HbA1c ≥ 7.5% who have completed an 18-month randomized controlled trial (RCT) on 90 g/day low-carbohydrate diet (LCD) were recruited and followed for one year. A three-day weighted food record, relevant laboratory tests, and medication effect score (MES) were obtained at the end of the previous trial and one year after for a total of 30 months period on specific diet.

**Results:**

71 (83.5%) patients completed the study, 35 were in TDD group and 36 were in LCD group. Although the mean of percentage changes in daily carbohydrate intake was significantly lower for those in TDD group than those in LCD group (30.51 ± 11.06% vs. 55.16 ± 21.79%, *p* = 0.0455) in the period between 18 months and 30 months, patients in LCD group consumed significantly less amount of daily carbohydrate than patients in TDD group (131.8 ± 53.9 g vs. 195.1 ± 50.2 g, *p* < 0.001). The serum HbA1_C_, two-hour serum glucose, serum alanine aminotransferase (ALT), and MES were also significantly lower for the LCD group patients than those in the TDD group (*p* = 0.017, *p* < 0.001, *p* = 0.017, and *p* = 0.008 respectively). The mean of percentage changes of HbA1_C_, fasting serum glucose, 2 h serum glucose, as well as serum cholesterol, triglyceride, low-density lipoprotein, ALT, creatinine, and urine microalbumin, however, were not significantly different between the two groups (*p* > 0.05).

**Conclusions:**

The one-year follow-up for patients on 90 g/d LCD showed potential prolonged and better outcome on glycaemic control, liver function and MES than those on TDD for poorly controlled diabetic patients.

## Introduction

Type 2 diabetes mellitus (DM) is an important lifestyle disease that initiates cardiovascular disease and frailty [1]. Although most diabetic organizations recommend 50–60% of total energy intake by carbohydrate and less than 30% of total energy intake by fat (with restrictions on saturated and transfat intake), some diabetic organizations recommend a personalized carbohydrate intake and accept low carbohydrate diet (LCD) as an option for diabetic control [2].

The LCD was defined as <130 g/d carbohydrate or <26% of total energy intake from carbohydrate for a diet of 2000 Kcal/day, 130 to 225 g/d carbohydrate was defined as moderate carbohydrate intake and over 225 g/day as high carbohydrate intake [3]. Type 2 diabetes patients on LCD are mostly very low in carbohydrate (VLCD, <50 g carbohydrate/day) [4, 5]. More and more evidences suggest the effectiveness of a LCD on weight and glycaemic control for type 2 diabetic patients [6, 7]; however, previous study also indicated the improvements of HbA1c and body weight may be short term effectiveness only [8].

The advantages of LCD are related to the degree of carbohydrate restriction, VLCD is more effective than standard low carbohydrate diets [9]. VLCD can control glycaemia and decrease body weight for up to 6 months in people with obesity and diabetes. VLCD is not recommended for long-term use based on current evidences show most patients are lack of adherence to strict carbohydrate restriction [10]. In considering the diet effectiveness and quality of life, moderate LCD is more acceptable.

We previously conducted an 18-month RCT of daily moderate LCD (≦90 g/day of carbohydrate) on type 2 diabetic patients aged 20 to 80 years with normal or obese body mass index (BMI). The results showed LCD render better glycaemic control with decreased medication effect score (MES), lower blood pressure, decreased body weight and waist and hip circumference without adverse effects on serum lipid profile, ALT, creatinine, and urine microalbumin than traditional diabetic diet (TDD) for type 2 diabetic patients [11]. The long-term effects of moderate LCD on glycaemic control, lipid status and the risk of atherosclerosis in type 2 diabetes patients are yet to be determined,

To investigate if diabetic patients can integrate the moderate LCD life style with their health belief and behavior after participating in a clinical trial, we conducted this study for 1-year follow-up after the completion of an 18-months moderate LCD RCT. The diet adherence and outcome were determined by three-day weighted food records, relevant laboratory tests, and medication effect score (MES).

## Research design and methods

### Study design

This was a 1-year follow-up study after an 18-month 90 g/day LCD RCT in type 2 diabetic patients. The previous RCT, a parallel-designed, single-center, open-label RCT study was published elsewhere [11].

This follow-up study was approved by the Human Research Ethics Committee of National Taiwan University Hospital (201712167RINC) and was conducted at the Department of Family Medicine, National Taiwan University Hospital from January 2018 to January 2019. All patients signed written informed consent prior to participation of the study.

### Study population and dietary intervention of the previous 18-month RCT

The study population and dietary intervention have previously been described in detail [11]. They were 92 type 2 diabetic adults. Age 20–80, with a diabetic history for over 1 year, with or without medical treatment, HbA1c ≧ 7.5% in the previous three months. The exclusion criteria included history of chronic kidney disease with serum creatinine ≧1.5 mg/dl; ALT more than three times above high normal; unstable angina or heart failure; frequent attack of gouty arthritis; current dietary changes due to eating disorder or on weight-reducing program; pregnant or lactating women.

For the LCD group, the daily carbohydrate intake was limited to less than 90 g (six servings of carbohydrates) without a restriction of total energy.

For the TDD group, the total daily calorie intake was stratified by individual BMI. It was decided by ideal body weight in kilogram times 20 (kcal/kg) for those with BMI > 24, times 25 (kcal/kg) for BMI between 18.5 and 24, and times 30 (kcal/day) for BMI < 18.5. Their macronutrient distribution was 50–60% of carbohydrates, 1.0–1.2 g/kg of protein and ≦30% of fat.

### Surveillance of the diet during the one-year of follow-up

At the completion of the previous 18-month RCT study, 85 (92.4%) patients agreed to continue on their diet in the following one year and have regular visits in 3 months’ interval.

A three-day weighted food record was collected at the end of the previous RCT and again 1 year later. The food record was calculated by a blinded evaluator using E-Kitchen nutritional analysis software.

### Assessment of the use of diabetic medication during the 1-year of follow-up

The use of diabetic medication was assessed by types (category by drug mechanism), numbers (total number of oral hypoglycemic agents and injected insulins) and MES (medication effect score).

The MES was the overall utilization of antiglycaemic agents. It was calculated by the sum of median absolute reduction of HbA1c times the percentage of the maximum daily dose for each medication, including insulin [12, 13]. The higher the MES indicated the greater use of medication.

The assessment was done at the beginning and the end of the 18-month RCT study and again at the 30 months (1-year follow-up).

### Outcome measurement

The primary outcomes were glycaemic control status and change in MES. The secondary outcomes were changes in serum lipid profiles, ALT, creatinine, and urine microalbumine.

Overnight fasting blood samples was collected and analysed for fasting glucose, HbA1c, total cholesterol, triglyceride, low density lipoprotein (LDL), high-density lipoprotein (HDL), creatinine, ALT and microalbumin/cre excretion, 2 h postprandial blood samples were collected and analysed for PC glucose and at the end of 18-month RCT study and again at the 30 months (1 year follow-up).

Blood pressure and anthropometric measurements, including weight, BMI, waist. hip and thigh circumference, as well as body fat content were collected at the end of 18-month RCT study and again 12 months then after (1-year follow-up). The method of measurement in detail was available in the previously published LCD RCT study [11].

For the convenience of viewing, the two observational points (at the end of the 18-month RCT and at 1 year follow-up) were reported as “at” 18 and 30 months. The difference of mean of percentage change between 18 and 30 months was reported as “the mean of percentage change”.

### Statistics

The results analysis of the follow-up study was conducted by using a per protocol analysis. A paired *t*-test was conducted to compare the mean change at 18 months and 30 months within the TDD and LCD groups regarding nutrient components, glycaemic control, lipid profiles, ALT, creatinine, microalbumin/cre excretion, blood pressure, anthropometric measurements and diabetic medication usage. An independent *t*-test was used to compare the differences at separate time points (18 and 30 months) and for the mean of percentage change (from 18 to 30 months) between the TDD and the LCD groups. The time trend of glycaemic control, MES, weight, blood pressure, and lipid profiles between the TDD group and the LCD group were analysed by a generalized estimating equations (GEE) method with an autoregressive (AR) covariance matrix. All analyses were conducted using SAS statistic software package 9.4 version (TS1M3 DBCS3170). A *p*-value < 0.05 was deemed statistically significant.

## Results

A total of 71 (83.5%) patients completed the 1-year follow-up study, including 35 (83.3%) in the TDD group and 36 (83.7%) in the LCD group. Seven withdrawers from TDD group and seven withdrawers from LCD group including one cardiovascular death in each group. There were no significant differences between groups in their baseline demographics, nutritional characteristics, laboratory results, anthropometric measurements, diabetic medication and use of lipid-lowering agents (*p* > 0.05, Tables 1–3).

**Table 1:** Characteristic of the patients followed at one year after 18-month intervention trial.

Characteristics	TDD (*n* = 35)		LCD (*n* = 36)		*p*-value
Age (years)	63.2 ±	6.8	63.3 ±	10.9	0.951
Age < 65	20	57.10%	16	44.40%	
Age ≥ 65	15	42.90%	20	55.60%	
Sex (female)	23	65.70%	23	63.90%	0.872
Education (years)	10.7 ±	4.5	11.5 ±	4.1	0.459
Marital status (single or widow)	7	20%	12	33.30%	0.205
Smoker	9	25.70%	8	22.20%	0.73
Alcohol use	12	34.30%	12	33.40%	0.932
BMI ≥ 24(kg/m^2^)	25	71.40%	29	80.60%	0.368
Hypertension	26	74.30%	26	72.20%	0.844
Duration of diabetes (years)	10	±7.2	10.4 ±	8	0.819
Family history of diabetes	25	71.40%	28	77.80%	0.539
Diabetic treatment					
No medication	1	2.90%	2	5.60%	0.674
OHA	28	80%	30	83.30%	
Insulin or (OHA with insulin)	6	17.10%	4	11.10%	
Statin medication	18	51.40%	17	47.20%	0.723
Fibrate medication	3	8.60%	0	0	0.115

*TDD* traditional diabetic diet, *LCD* low carbohydrate diet, *OHA* oral hypoglycaemic agents.

Data were mean ± SD or *n* (%); independent *t*-test or chi-square test (and Fisher’s exact test if *n* < 5).

**Table 2:** Data of baseline, 18 months, and 30 months for patients in the traditional diet and the low-carbohydrate diet.

Characteristics	Group	Baseline	18 months	^a^*p*-value	30 months	^b^*p*-value	Within group	Mean of percentage change	Between group
							^c^*p*-value		^d^*p*-value
Carbohydrate (g/d)	TDD	234.9 ± 54.3	151.1 ± 29.8	<0.001*	195.1 ± 50.2*	<0.001*	<.0001*	30.51 (19.45 ~ 41.57)	0.0455*
	LCD	240.6 ± 75.1	88.0 ± 29.9		131.8 ± 53.9*		<.0001*	55.16 (33.37 ~ 76.95)	
Protein (g/d)	TDD	72.9 ± 26.8	72.0 ± 18.5	0.018*	60.5 ± 19.4	0.153	0.0037*	−14.33 (−23.37 ~ −5.29)	0.9055
	LCD	71.4 ± 18.7	82.4.3 ± 22.1		68.2 ± 24.5		0.0011*	−15.13 (−25.33 ~ −4.93)	
Fat (g/d)	TDD	65.6 ± 26.0	67.2 ± 22.2	0.16	45.4 ± 17.3	0.299	<.0001*	−0.22 (−2.57 ~ 2.13)	0.9604
	LCD	58.3 ± 25,7	73.1 ± 16.9		49.5 ± 16.0		<.0001*	−0.12 (−3.58 ~ 3.34)	
Polyunsaturated fat (g/d)	TDD	10.2 ± 5.7	8.8 ± 3.4	0.047*	7.9 ± 4.2	0.956	0.5692	10.08 (−16.56 ~ 36.72)	0.4907
	LCD	9.4 ± 5.1	9.9 ± 7.2		7.8 ± 3.5		0.0254*	−1.83 (−24.55 ~ 20.89)	
Monounsaturated fat (g/d)	TDD	12.4 ± 7.3	12.2 ± 4.9*	0.008*	10.6 ± 6.1	0.144	0.297	1.92 (−24.31 ~ 28.15)	0.9293
	LCD	12.7 ± 7.3	17.4 ± 7.8*		12.7 ± 5.8		0.0191*	3.65 (−25.78 ~ 33.08)	
Saturated fat (g/d)	TDD	13.5 ± 8.3	9.9 ± 3.7*	0.009*	10.1 ± 5.9	0.434	0.8443	17.44 (−13.15 ~ 48.03)	0.1495
	LCD	13.8 ± 6.7	13.9 ± 8.5*		11.2 ± 5.1		0.0133*	−8.39 (−27.25 ~ 10.47)	
Energy (Kcal/d)	TDD	1779.2 ± 474.0	1461.0 ± 251.0	0.521	1409.6 ± 352.9	0.023*	0.3629	−2.58 (−10.35 ~ 5.20)	0.058
	LCD	1747.1 ± 468.4	1418.7 ± 290.8		1221.2 ± 325.2		0.0007*	−12.53 (−19.61 ~ −5.46)	

*TDD* traditional diabetic diet (*n* = 35), *LCD* low carbohydrate diet (*n* = 36).

Data were mean ± SD *p < 0.05.

^a^*p*-value: The difference between the TDD and LCD group at 18 months by an independent *t*-test.

^b^*p*-value: The difference between the TDD and LCD group at 30 months by an independent *t*-test.

^c^*p*-value: The mean of percentage change from 18 to 30 months within groups by a paired *t*-test.

^d^*p*-value: The mean of percentage change from 18 to 30 months between groups by an independent *t*-test.

**Table 3:** Changes of results of biochemistry tests, anthropometric and diabetic medications at the end of the 18-month intervention trial and 1 year after.

Characteristics	Group	Baseline	18 months	Between group	30 months	Between group	Within group	Mean of percentage change	Between group
				^a^*p* value		^b^*p* value	^c^*p* value		^d^*p* value
Glycemic control									
HbA1c (%)	TDD	8.5 ± 0.9	7.6 ± 1.0	0.000*	7.7 ± 1.0	0.017*	0.519	1.80 (−1.87 ~ 5.46)	0.2417
	LCD	8.4 ± 0.9	6.9 ± 0.6		7.2 ± 0.8		0.006*	22.12 (7.44 ~ 36.80)	
Fasting glucose (mg/dl)	TDD	159.5 ± 33.8	154.9 ± 37.1	0.008*	142.8 ± 44.7	0.579	0.168	−4.12 (−14.79 ~ 6.54)	0.2206
	LCD	160.6 ± 44.7	133.6 ± 27.9		137.3 ± 38.6		0.531	8.22 (−0.01 ~ 16.46)	
2-h glucose (mg/dl)	TDD	227.8 ± 56.8	215,1 ± 73.5	0.000*	217.3 ± 67.8	0.000*	0.881	9.52 (−4.78 ~ 23.81)	0.2161
	LCD	227.2 ± 65.4	133.8 ± 25.2		161.5 ± 58.7		0.005*	31.38 (11.24 ~ 51.51)	
Lipids									
Cholesterol (mg/dl)	TDD	177.0 ± 32.7	169.5 ± 29.6	0.588	174.5 ± 34.6	0.269	0.463	5.30 (−3.38 ~ 13.97)	0.6207
	LCD	179.3 ± 34.4	173.±32.0		184.2 ± 38.3		0.078	8.22 (−0.01 ~ 16.46)	
Triglyceride (mg/dl)	TDD	179.2 ± 118.5	163.1 ± 88.3	0.052	154.5 ± 54.0	0.779	0.625	8.82 (−5.48 ~ 23.11)	0.0683
	LCD	156.3 ± 70.7	128.0 ± 59.0		160.3 ± 107.4		0.044*	31.38 (11.24 ~ 51.51)	
LDL (mg/dl)	TDD	105.7 ± 25.8	101.4 ± 26.7	0.618	101.2 ± 31.1	0.832	0.966	4.12 (−9.26 ~ 17.50)	0.6314
	LCD	101.8 ± 27.2	98.3 ± 26.8		102.8 ± 33.6		0.301	8.50 (−4.23 ~ 21.23)	
HDL (mg/dl)	TDD	44.6 ± 9.8	45.9 ± 8.3		49.7 ± 14.5		0.09	9.13 (−0.62 ~ 18.89)	0.0391*
	LCD	47.4 ± 10.3	52.5 ± 11.8	0.008*	51.5 ± 13.6	0.588	0.376	−1.97 (−6.18 ~ 2.24)	
Other laboratory									
Creatinine (mg /dl)	TDD	0.82 ± 0.28	0.87 ± 0.31	0.759	0.95 ± 0.39	0.742	0.068	9.89 (−2.72 ~ 22.50)	0.3109
	LCD	0.86 ± 0.24	0.89 ± 0.23		0.92 ± 0.27		0.124	3.22 (−0.71 ~ 7.15)	
ALT (mg/dl)	TDD	24.4 ± 12.8	22.8 ± 14.8	0.017*	20.6 ± 11.5	0.017*	0.302	0.82 (−10.90 ~ 12.55)	0.7727
	LCD	23.5 ± 16.5	16.1 ± 5.6		16.5 ± 10.57		0.838	3.85 (−13.77 ~ 21.46)	
Microalbumin/cre (U)	TDD	0.09 ± 0.17	0.11 ± 0.26	0.659	0.19 ± 0.44	0.278	0.254	192.56 (51.48 ~ 333.64)	0.2407
	LCD	0.32 ± 0.88	0.14 ± 0.37		0.36 ± 0.79		0.07	2815.45 (−1643.53 ~ 7274.42)	
Blood pressure									
Systolic (mmHg)	TDD	130.5 ± 12.3	132.4 ± 11.5	0.000*	129.4 ± 14.1	0.419	0.167	−2.06 (−5.32 ~ 1.20)	0.0013*
	LCD	131.8 ± 13.2	123.1 ± 9.9		132.2 ± 15.4		0.005*	8.06 (2.95 ~ 13.18)	
Diastolic (mmHg)	TDD	74.9 ± 9.1	76.3 ± 9.9	0.018*	74.7 ± 101	0.782	0.434	−0.89 (−6.44 ~ 4.66)	0.0619
	LCD	76.4 ± 9.9	71.4 ± 7.0		75.4 ± 9.6		0.05	6.57 (0.83 ~ 12.31)	
Anthropometric									
Body weight (kg)	TDD	67.5 ± 11.6	66.7 ± 12.0	0.712	66.2 ± 12.7	0.662	0.291	−0.82 (−2.31 ~ 0.67)	0.6036
	LCD	70.2 ± 14.8	67.8 ± 13.3		67.6 ± 13.4		0.551	−0.32 (−1.57 ~ 0.92)	
BMI (kg/m^2^)	TDD	26.5 ± 3.4	25.9 ± 2.9	0.392	25.7 ± 3.1	0.399	0.239	−0.89 (−2.37 ~ 0.59)	0.8923
	LCD	27.7 ± 4.8	26.7 ± 4.4		26.5 ± 4.4		0.199	−0.76 (−2.01 ~ 0.49)	
Fat (%)	TDD	35.0 ± 7.9	35.4 ± 7.3	0.813	35.2 ± 7.0	0.875	0.605	−0.22 (−2.57 ~ 2.13)	0.9604
	LCD	36.7 ± 8.1	35.9 ± 8.5		35.5 ± 7.7		0.437	−0.12 (−3.58 ~ 3.34)	
Waist circumference (cm)	TDD	93.4 ± 8.2	91.2 ± 7.8	0.341	91.0 ± 8.7	0.266	0.684	−0.27 (−1.48 ~ 0.94)	0.6173
	LCD	94.6 ± 12.1	89.3 ± 9.2		88.6 ± 9.2		0.25	−0.71 (−2.00 ~ 0.59)	
Hip circumference (cm)	TDD	99.2 ± 7.9	95.6 ± 6.9	0.664	95.4 ± 7.2	0.559	0.569	−0.23 (−1.07 ~ 0.60)	0.5894
	LCD	100.9 ± 10.3	94.9 ± 7.6		94.3 ± 7.8		0.174	−0.55 (−1.36 ~ 0.27)	
Thigh circumference (cm)	TDD	50.7 ± 7.3	45.7 ± 3.8	0.411	45.7 ± 4.1	0.205	0.908	0.08 (−1.02 ~ 1.19)	0.2733
	LCD	50.4 ± 7.0	44.9 ± 4.5		44.4 ± 4.4		0.162	−0.86 (−2.21 ~ 0.48)	
Diabetic medication									
Types of medications	TDD	2.7 ± 1.1	2.6 ± 1.0	0.261	2.7 ± 0.9	0.162	0.057	13.81 (2.17 ~ 25.45)	0.5342
	LCD	2.6 ± 1.6	2.3 ± 1.4	0.042*	2.4 ± 1.2	0.158	0.201	8.33 (−5.27 ~ 21.93)	
Number of medications	TDD	5.9 ± 3.7	5.7 ± 3.4		5.5 ± 3.0		0.971	8.85 (−8.61 ~ 26.31)	0.2692
	LCD	5.6 ± 4.2	4.1 ± 3.3		4.5 ± 3.0		0.581	23.40 (3.25 ~ 43.54)	
MES	TDD	2.2 ± 1.4	2.1 ± 1.1	0.003*	2.3 ± 1.0	0.009*	0.127	19.92 (1.93 ~ 37.90)	0.513
	LCD	1.9 ± 1.4	1.4 ± 1.0		1.6 ± 1.0		0.030*	29.17 (6.69 ~ 51.65)	

*TDD* traditional diabetic diet (*n* = 35), *LCD* low carbohydrate diet (*n* = 36), *LDL* low density lipoprotein, *HDL* high density lipoprotein, *ALT* alanine aminotransferase, *BMI* body mass index, *MES* medication effect score.

Data were mean ± SD *p < 0.05.

^a^*p*-value: The difference between the TDD and LCD group at 18 months by an independent *t*-test.

^b^*p*-value: The difference between the TDD and LCD group at 30 months by an independent *t*-test.

^c^*p*-value: The mean of percentage change from 18 to 30 months within groups by a paired *t*-test.

^d^*p*-value: The mean of percentage change from 18 to 30 months between groups by an independent *t*-test.

### Changes in diet

At 18 months, the LCD group consumed a significant lower amount of carbohydrate but higher amount of protein, saturated and monounsaturated fat than the TDD group (*p* < 0.05) (Table 2). Although both groups had significant increase amount of their carbohydrate intake from 18 to 30 months, the LCD group had significantly higher mean of percentage change for carbohydrate than the TDD group (*p* < 0.05) during the same period of time, and the LCD group consumed lower carbohydrate than the TDD group at 30 months (*p* < 0.05).

In the LCD group at 18 months, there were 20 (57.1%) patients taking carbohydrate <90 g/day, 10 (28.6%) patients taking carbohydrate between 90 to 130 g/day, and 5 (14.3%) patients were in the moderate carbohydrate intake (130–225 g/day). At 30 months, there were 5 (13.9%) patients in <90 g/day LCD, 15 (41.7%) patients in LCD between 90 g and 130 g/day, 13 (36.4%) patients in moderate carbohydrate intake. Two (5.6%) patients shifted to TDD and 1 (2.8%) patient quit LCD with no dieting.

Patients in both groups decreased protein and fat intake significantly from 18 to 30 months (*p* < 0.05), and there was no significant difference of mean of change percentages for protein and fat between the groups.

The daily intake of polyunsaturated, monounsaturated and saturated fat in the LCD group was significantly higher than those in the TDD group at 18 months (*p* < 0.05). From then on, consumption of those nutrients significantly decreased in the LCD group (*p* < 0.05) but remained stable in the TDD group from 18 to 30 months. However, the mean of percentage changes of daily intake of polyunsaturated, monounsaturated and saturated fat were not significantly different between the LCD and TDD groups in this period of time (Table 2).

Although the LCD group consumed a significantly lower total daily calorie than the TDD group from 18 to 30 months (*p* < 0.05) (Table 2), there was no significant difference of mean of percentage change for total daily calorie intake between two groups.

### Changes in glycaemic control

**Fig. 1:**
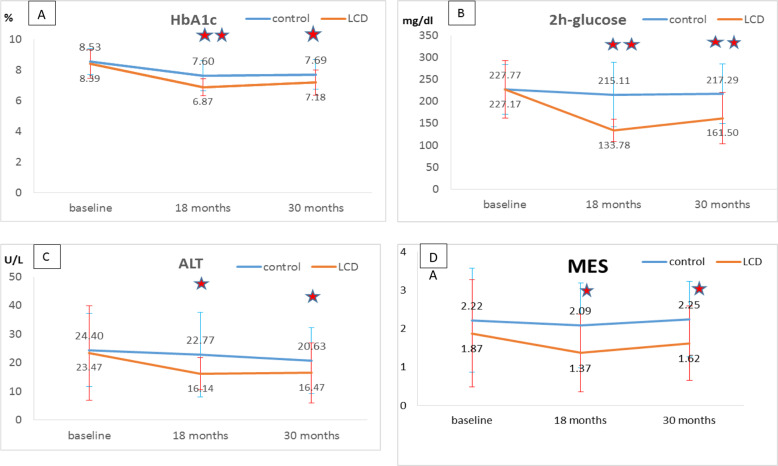
Baseline, 18-month and one-year (30-month) follow-up across dietary intervention groups. HbA1c (**A**), 2 h glucose (**B**), ALT (**C**), and MES (**D**) were significantly different between the TDD and LCD groups (*p < 0.05; **p < 0.001). MES medication effect score, ALT alanine aminotransferase, TDD traditional diabetic diet, LCD low carbohydrate diet.

The HbA1c and 2 h of post prandial serum glucose were significantly lower for the LCD group than the TDD group at both 18 and 30 months (*p* < 0.05 for both) (Fig. 1A, B), but the HbA1c and 2 h of post prandial serum glucose was significantly increased in the LCD group (*p* < 0.05) from 18 to 30 months. No significant difference was observed for fasting serum glucose between the groups. The mean of percentage changes for HbA1c, fasting serum glucose, and 2 h of post prandial serum glucose from 18 to 30 months was not significantly different between the TDD and LCD groups (Table 3).

### Changes in lipid profile

For patients in the LCD group, the triglyceride level significantly increased (*p* < 0.05) and the mean of percentage change for HDL level significantly decreased (*p* < 0.05) from 18 to 30 months. However, the mean of percentage changes for total cholesterol, triglyceride, and LDL were not significantly different between the TDD and LCD groups in this period of time (Table 3).

The patient number of statin use was observed to decreased from 18 (51.4%) to 16 (45.7%) in TDD group but increased from 17 (47.2%) to 18 (50%) in LCD group at 18 months in our previous RCT study. At 30 months, the patient number of statin use was up to 25 (71.4%) in TDD group and was significantly higher than those in LCD group which had decreased to 17 (47.2%) (*p* = 0.038). Despite medication intervention and adjustment, changes of lipid profile showed no significant difference between two groups at 18 and 30 months (Table 4).

**Table 4:** Change of lipid profile at the end of 18-month intervention and one year after adjusted with statin and fibrate.

	Δ(18 months—baseline)		Δ(30 months—baseline)		Group × time interaction
	Mean	(SD)	Mean	(SD)	*p*-value
Total cholesterol (mg/dl)					
TDD	−4.93	(35.10)	−2.43	(44.46)	0.5299
LCD	−5.23	(37.46)	4.83	(45.60)	
Triglyceride (mg/dl)					
TDD	−17.81	(95.42)	−24.69	(115.98)	0.0747
LCD	−31.40	(78.49)	3.92	(98.44)	
LDL (mg/dl)					
TDD	−3.03	(25.32)	−4.52	(33.73)	0.4857
LCD	−1.77	(29.63)	1.03	(33.04)	
HDL (mg/dl)					
TDD	2.94	(9.94)	5.15	(14.02)	0.1082
LCD	4.94	(8.24)	4.11	(8.84)	

Estimated by generalized estimating equation.

Adjusted statin, fibrates.

*TDD* tradtitional diabetic diet, *LCD* low carbohydrate diet, *SD* standard deviation.

### Changes in other laboratory profiles

Compared to at 18 months, the serum creatinine, ALT and urine microalbumin were not significantly different within the TDD and LCD group at 30 months. The serum ALT levels was significantly lower in the LCD group than in the TDD group at 18 and 30 months (*p* < 0.05) (Fig. 1C). The mean of percentage changes of serum creatinine, ALT, and urine microalbumine were not significantly different between the TDD and LCD groups from 18 to 30 months (Table 3).

### Changes in blood pressure

The patients in LCD group had significantly higher systolic blood pressure (SBP), diastolic blood pressure (DBP) and the mean of percentage change of SBP than those in the TDD group (*p* < 0.05, *p* = 0.050, and *p* < 0.05 respectively) from 18 to 30 months (Table 3).

The use of antihypertensive drugs was slightly increased (losartan 25 mg added) for 1 (2.8%) patient in LCD group, and slightly increased (valsartan 80 mg added) in 1 (2.9%) patient and slightly decreased (losartan 25 mg reduction) in 1 (2.9%) patient in TDD group. All had no statistic significance.

No participant reported getting hormone replacement therapy during the study period.

### Changes in anthropometric measurements

The body weight, BMI, body fat content, waist, hip, and thigh circumferences for patients at 30 months were not significantly different than the same person at 18 months in the TDD and LCD groups (*p* > 0.05). The mean of percentage changes in body weight, BMI, body fat content, waist, hip, and thigh circumferences were not significantly different between patients in the TDD and LCD groups from 18 to 30 months (Table 3).

### Changes in use of diabetic medication

Compared the same patient at 18 and 30 months, the MES was significantly increased for those in the LCD group (*p* < 0.05). However, the LCD group patients had significantly lower MES at 18 months and 30 months than the TDD group patients (*p* < 0.05) (Fig. 1D).

The mean of percentage changes in MES and the types and number of diabetic medications were not significant between patients in the TDD and LCD groups from 18 to 30 months (Table 3).

## Discussion

LCD has been proven to be beneficial for glycaemic control on type 2 Diabetes patients but not for its long term effects [3, 14]. Our previous study had proven a better outcome of the 90 g/day LCD over TDD for poorly controlled type 2 Diabetes patients in 18 months [11]. This follow-up study focused on the adherence of LCD and the long term outcomes in different aspect for the same group of patients.

In our study, although the mean of percentage change for carbohydrate intake was higher and daily carbohydrate intake increased in the LCD group, the goal to remain on ≦130 g/day of carbohydrate diet is achievable. Patients on LCD had better outcome than those on TDD in many aspects including taking significantly less amount of carbohydrate, better HbA1c and post prandial serum glucose level, and better MES.

Although the carbohydrate intake increased for patients in both LCD and TDD groups and the mean of percentage changes of carbohydrate was significant higher in patients in LCD group at 30 months than the same individual at 18 months, the LCD group patients had significantly lower HbA1c and two hours postprandial serum glucose level than the TDD group patients at 30 months. This result indicated potential prolonged effects in glycaemic control by LCD.

The previous study [14] showed no difference of high carbohydrate intake for their TDD and LCD group at 1-year follow-up after 6-month 130 g/day LCD RCT. The 1-year follow-up carbohydrate intake in their LCD group was no more fulfilled the LCD criteria of <130 g/day [3]. Therefore, the beneficial effect of the 130 g/day LCD on reduction of HbA1c and BMI did not persist in comparison with TDD 1 year after [14].

A recent systematic review indicated patient adherence to a ketogenic LCHF (low carb high fat) diet may be difficult [15]. Other studies on 80–90 g/day LCD for 6 months with 22 and 44 months follow-up showed beneficial effects on body weight and serum HbA1c level [16, 17], but those studies failed to provide detail information on daily intake of carbohydrate and total calorie. Our patients had slightly increased in their carbohydrate intake and had decreased their intake of protein and all kinds of fat (saturated, unsaturated, and trans-fatty acid). The less total daily calorie intake from substituting fat with carbohydrate in the LCD group at 30 months resulted in non-significantly reducing weight, BMI and other anthropometric measurements despite increasing carbohydrate intake and HbA1c. The LCD group continues to have better glycaemic control than the TDD group at 30 months. We concluded, when patients take a LCD program, they should be very strict on their diet and achieved a lowest possible HbA1c level to begin with, so in the long run, even if they have slight increase in their carbohydrate intake, they can maintain good glycaemic control. By this arrangement, patients may be adherent to their LCD for longer period of time and have prolong benefits from their glycaemic control. Although patients may not be able to reduce their body weight or BMI further, they may be more satisfied with their life quality and remain good outcome from LCD at the same time.

Patients in LCD group had a significantly lower MES but they had better HbA1c, this was another indication for the benefits of LCD on Diabetes patients. Since patients are taking fewer medications, this will provide further benefits to the patients if polypharmacy is a concern.

In a meta-analysis, the beneficial effects of LCD on the liver enzymes of non-alcoholic fatty liver disease (NAFLD) patients was contradictory and showed had negative results [18]. However, those clinical trials were heterogeneous in their definitions for LCD, their effectiveness on the liver enzymes were not conclusive. In a recent review of dietary strategies targeting intrahepatic fat in NAFLD [19], the study cited the effectiveness of carbohydrate restriction on liver fat was not related to body weight loss [20]. In our study, the improvement of ALT remained despite weight gain was observed in LCD group with daily carbohydrate ≦ 130 g/day.

Studies have shown patients on Mediterranean diet or LCD have prolonged favorable outcomes on their serum total cholesterol and triglyceride level even if they have gained weight [21]. However, serum triglyceride level in our patients had elevated. This result is probably due to our LCD patients had increased intake of carbohydrates.

Our study has several limitations. Firstly, patients were from the same department of a single medical center, the application of study results should be with caution. Secondly, the diagnosis of type 2 diabetes was made by clinicians based on patients’ serum glucose level and HbA1c, no other diagnostic tests were performed. Thirdly, 20–30% patients in both groups had BMI less than 24, so the effectiveness of LCD on weight loss and other anthropometric measurements may be undermined. Fourthly, our patients were not screened for NAFLD, there may be other explanations for the changes of ALT. Fifthly, patients were not measured for their physical activity. However, there was no significant difference observed in this measurement between patients in different group in the previous study [11]. There are also several strengths for this study. The missing number was small, comprehensive outcome measurement included MES and urine microalbumin, the follow up period was longer than one year.

In summary, the 90 g/day LCD is an effective and feasible choice for diet control in type 2 diabetes patients. It has potential long term effects with better glycaemic control, lower ALT and MES than TDD for poorly controlled type 2 diabetic patients.

## Data Availability

The data that support the findings of this study are available from the corresponding author upon reasonable request.
